# Terahertz Time Domain Spectroscopy of Transformer Insulation Paper after Thermal Aging Intervals

**DOI:** 10.3390/ma11112124

**Published:** 2018-10-29

**Authors:** Liang Wang, Chao Tang, Shiping Zhu, Shengling Zhou

**Affiliations:** College of Engineering and Technology, Southwest University, Chongqing 400715, China; 18227589426@163.com (L.W.); zhu_s_p@sina.com (S.Z.); swuzhousl@163.com (S.Z.)

**Keywords:** novel diagnostic technique, terahertz-time-domain spectroscopy, transformer insulation paper, DFT, molecular dynamics simulation

## Abstract

An accelerated thermal aging process was used to simulate the condition of paper insulation in transformer oil-paper systems. Optical parameters of the insulation paper after various aging intervals were analyzed with terahertz time-domain spectroscopy (THz-TDS) over the range 0.1~1.8 THz. The result shows that the paper had seven absorption peaks at 0.19, 0.49, 0.82, 1.19, 1.43, 1.53, and 1.74 THz, and density functional theory of B3LYP/6-311G+ (d, p) was used to simulate the molecular dynamics of the repeating component (cellobiose) of the cellulose paper. Theoretical spectra were consistent with experiment, which had absorption peaks at 0.18, 0.82, 1.47, and 1.53 THz in the same frequency range. At the same time, the paper samples after various aging intervals had different refractive indexes, and least squares fitting revealed a linear relationship between the degree of polymerization and the refractive index of the paper. Hence, this paper demonstrates that THz-TDS could be used to analyze the aging condition of transformer insulation paper and provides the theoretical and experimental basis for detection.

## 1. Introduction

Terahertz (THz) radiation has wavelengths of 30 μm~3 mm (0.1~10 THz) between far-infrared and microwaves. It is a transitional region between macro-electronics and micro-photonics [1,2]. And as a cutting-edge analytical technique, terahertz (THz) spectroscopy has multivariate characteristics, such as fingerprint absorption, coherence, and low ionization damage [3,4]. Furthermore, it is quite sensitive to different chemical composition and intermolecular interactions and oscillations of materials, and there are different fingerprint characteristics in the THz region [5,6]. Therefore, the THz absorption and dispersion frequency response of materials was used to obtain important information on molecular structures [7], including dielectrics materials [8], DNA [9], and pesticides [10]. Applications also include security [11] and nondestructive testing [12]. THz technology has a great scientific research and application potential, particularly with continuous development of THz emitters [13] and detectors [14]. And with the development of THz time-domain spectroscopy (THz-TDS) and THz imaging, there have been numerous breakthroughs in this field. These techniques are far beyond conventional tools for analyzing various materials [1]. However, there is no report on the application of THz technology to the detection and analysis of transformer insulation paper after various aging intervals.

The power transformer is the core equipment of power systems, and plays an extremely important role in it [15]. The safety operation of power transformers is of great significance to the stability of power systems. Because it will cause immeasurable losses, if an accident occurs [16]. However, the safe operation of the power transformer depends largely on the integrity of insulation components, so the study of the transformer insulation system has been highly valued by the power sector and experts [17]. According to the statistics, the power transformer accidents are mainly caused by the internal insulation faults [18]. And the main component of transformer internal insulation is insulating oil-paper. The paper is the primary solid insulation material in transformers, and is mainly composed of α-cellulose, a long-chain polymer of glucose monomers [19]. Aging of the insulation paper during transformer operation involves cellulose degradation. To a certain extent, the operating life of power transformers is based on that of the paper because it cannot be replaced [20]. Therefore, it is important to monitor the aging condition of the paper for safe operation. The traditional method is to detect the degree of polymerization of the paper, which is an effective criterion for the aging condition of insulation paper [21]. However, the shortcomings of this method are also obvious, it requires complicated pre-processing.

In this work, a traditional method was used to measure the degree of polymerization of insulation paper [22]. And then, to detect the spectra of the degraded transformer insulation paper, the spectral characteristics of thermally aged paper were analyzed by terahertz time-domain spectroscopy (THz-TDS). And the density functional theory (DFT) is a useful tool for studying the molecular structure and vibrational modes of molecules, which has been applied to assign the specific absorption peaks of THz spectra [10]. Therefore, in this paper, the DFT of B3LYP/6-311G+(d, p) was applied to simulate the molecular dynamics for analyzing peaks of the repeating component of paper based on the isolated molecule. And according to the results of DFT molecular dynamics simulations, the experimental spectral absorption peaks were analyzed. This work lays a foundation for studying the terahertz spectrum parameters of transformer insulation paper after various aging intervals by using terahertz technology, and provides a theoretical and experimental basis for predicting the safe operation life of transformer.

## 2. Materials and Methods

### 2.1. Sample Preparation

The insulation oil was 25# ordinary mineral oil purchased from PetroChina Co., Ltd. (PetroChina Co., Ltd., Karamay, China), and the paper was 0.5-mm-thick kraft paper produced by Sichuan EM Technology Co., Ltd. (Sichuan EM Technology Co., Ltd., Mianyang, China). Before the experiments, drying and degassing of the oil-paper was necessary. First, the insulation paper was dried for 24 h at 343 K in a 500 Pa vacuum to keep the remaining water content less than 0.5%. Then, degassed insulating oil was heated to 313 K and injected into the container holding the insulation paper to produce an oil-paper ratio of 20:1. The oil-paper was then placed in a 50 Pa vacuum at 313 K for 24 h [23]. Finally, the samples were placed in an evacuated box to perform thermal aging at 403 K. Samples were removed at different aging intervals and THz optical analyses of the insulation paper were performed. And following that, the paper was in the state of solid sheet, and it was cut into a diameter of about 10 mm disc-shaped and sealed in a PE film during detecting. The surfaces of the paper were smooth and parallel to reduce the influence of scattering loss, as in Reference [24].

### 2.2. THz spectral Acquisition

**Figure 1:**
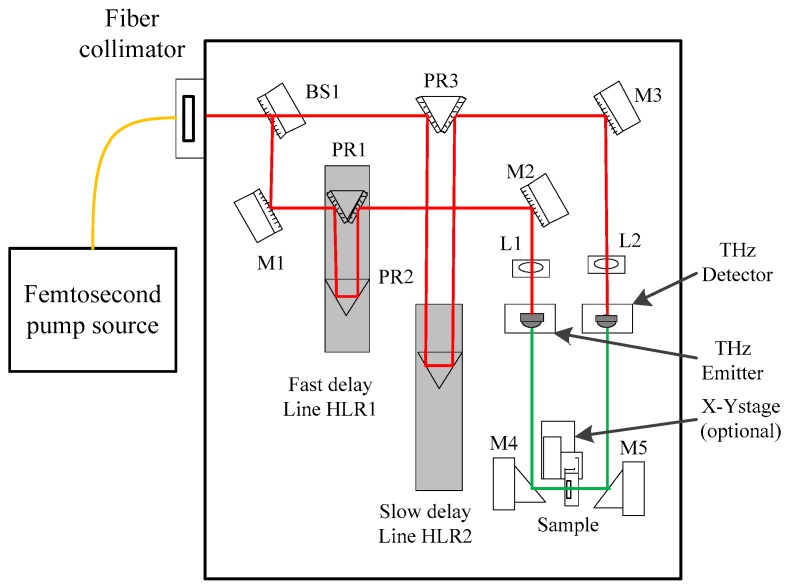
Optical path of terahertz time-domain spectroscopy system.

In this work, a T-Spec terahertz time domain spectrometer (Ekspla, Inc., Vilnius, Lithuania), along with a photoconducting antenna illuminated by ultrashort laser pulses, was used for THz radiation and detection. The pump laser provided 50~150 fs pulses at 1050 ± 40 nm wavelengths, with more than 40 mW of output power at an 80 MHz pulse repetition rate. And a THz emitter and detector with a micro strip antenna integrated with a low-temperature-grown GaBiAs photoconductor and a silicon lens mounted on the back side of a photoconductive antenna was used. The THz-TDS optical layout is illustrated in Figure 1. The experiment was carried out at room temperature of 296 K, and dry nitrogen was filled into the sample bin to avoid the effect of moisture. When acquisition of the THz spectrum, the THz pulses were focused on the surfaces of the samples vertically. And the average spectrum of 1024 time-domain scans was obtained as the spectrum of the test sample.

### 2.3. THz Data Processing

The Dorney [25] and Duvillaret [26] mathematical model of THz optical parameters was used to calculate the refractive index and the absorption coefficient of the insulation paper over the range 0.1~1.8 THz. This model requires that the response function of the THz time-domain system does not change with time, and the sample structure remain was uniform and two surfaces are parallel. The prepared samples in this work meet the above conditions.

By applying the fast Fourier transform (FFT) to the detected time-domain signals, the frequency-domain function E(ω) can be obtained. $E_{\mathrm{ref}}(\omega )$ and $E_{s}(\omega )$ were called the reference and sample signals, respectively. Therefore, the complex mapping function of the sample was expressed as Equation (1):

(1) $$H(\omega )=\frac{E_{\mathrm{ref}}(\omega )}{E_{s}(\omega )}=\frac{4N}{{\left(N+1\right)}^{2}}e^{i2\pi (N-1)d\omega /c}=\rho (\omega )e^{\varphi (\omega )}$$

Furthermore, κ(ω) is the extinction coefficient of sample, indicating its absorption characteristics. And N=n(ω)−jκ(ω) is the complex refractive index of sample. If the surface multiple reflection was neglected, and the extinction coefficient of the sample is much smaller than that of the refractive index. Then the formulas for calculating the refractive index n(ω), extinction coefficient κ(ω) and absorption coefficient α(ω) of the sample were deduced as Equation (2)–(4):

(2) $$n(\omega )=\varphi (\omega )\frac{c}{\omega d}+1=1+\frac{c}{\omega d}[\varphi_{s}(\omega )-\varphi_{ref}(\omega )]$$

(3) $$\kappa (\omega )=\frac{c}{\omega d}\ln[\frac{4n(\omega )}{\rho (\omega ){\left[n(\omega )+1\right]}^{2}}]=\frac{c}{\omega d}\ln\frac{\left|E_{ref}(\omega )\right|}{\left|E_{s}(\omega )\right|}$$

(4) $$\alpha (\omega )=\frac{2\kappa (\omega )\omega }{c}=\frac{2}{d}\ln[\frac{4n(\omega )}{\rho (\omega ){\left[n(\omega )+1\right]}^{2}}]=\frac{2}{d}\ln\frac{\left|E_{ref}(\omega )\right|}{\left|E_{s}(\omega )\right|}$$

where ω, c and d were frequency (Hz), light speed (m/s), and the thickness of the sample (m), while ρ(ω) and φ(ω) were amplitude ratio of reference and sample signals, and the phase difference between reference and sample signals, respectively.

## 3. Analysis and Discussion

### 3.1. THz Time-Domain Waveforms and Frequency-Domain Spectra

**Figure 2:**
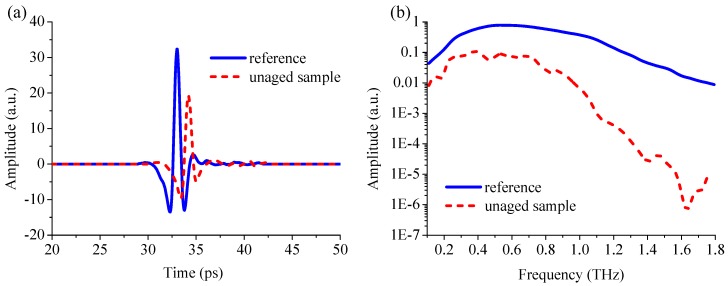
The THz spectra of reference and the unaged insulation paper. (**a**) The time-domain waveform; (**b**) The frequency-domain spectra.

The THz spectra of polyethylene (PE) and paper were measured by terahertz time-domain spectroscopy (THz-TDS) as the reference and sample spectra, respectively. PE had extremely low absorption of THz radiation and had no influence on the position of the absorption peaks of the paper, so it was used as a reference. Figure 2 shows the time-domain waveforms and the frequency-domain spectra of the reference and the unaged paper. To present better time-domain waveforms, the THz pulse time trace at 20–50 ps was shown in Figure 2a. It can be seen that the amplitude of the sample was attenuated and the time was delayed compared with the reference. The amplitude of the reference and sample signals was 32.342 and 19.229 a.u., respectively. This is due to the THz pulse was absorbed, reflected, and scattered on the surface of the sample. Compared with reference, the time delay of the paper was 1.174 ps. This is related to the difference of THz wave propagation velocity in the PE and paper. The frequency-domain spectra obtained by fast Fourier transform (FFT) of the time-domain spectrum (0–50 ps) were shown in Figure 2b. Compared with the reference, there are anomalous spectral pits in the frequency region of the paper. It implied that PE does not absorb the THz wave, and these pits can be assigned as the THz optical characteristics of the paper.

### 3.2. Analysis of Absorption and Refraction Characteristics

**Figure 3:**
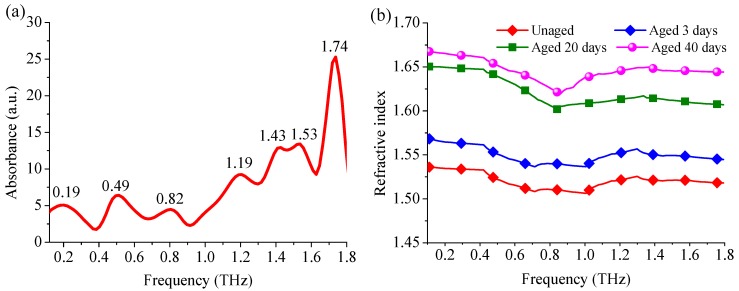
Optical parameters of transformer insulation paper. (**a**) Absorption coefficients of unaged paper; (**b**) Refractive indices of unaged paper and paper aged for 3, 20, and 40 days at 403 K.

The THz absorption and refraction characteristics of paper were calculated according to the formulas in Section 2.3. Figure 3 depicts the absorption coefficient and refractive indices of paper after thermal aging intervals from 0.1 to 1.8 THz. During data processing, it may be observed that the baseline of the absorption spectrum raises with the frequency increases. This may be due to the higher frequency, which enables the absorption and scattering of THz wave stronger. Furthermore, the low SNR may experience be interfered by from high frequency oscillation and multiple reflection noise. So, before data processing, the wavelet de-noise algorithm was applied to the signal analysis [27]. The result shows that the unaged paper has absorption peaks at 0.19, 0.49, 0.82, 1.19, 1.43, 1.53, and 1.74 THz, which were shown in Figure 3a. Figure 3b depicts that the average refractive index of the unaged insulation paper was 1.504, and those after 3, 20, and 40 days at 403 K were 1.550, 1.621, and 1.641, respectively. It reflected the absorption and dispersion characteristics of insulation paper after thermal aging intervals, and it can be used to evaluate the aging condition of insulation paper.

**Figure 4:**
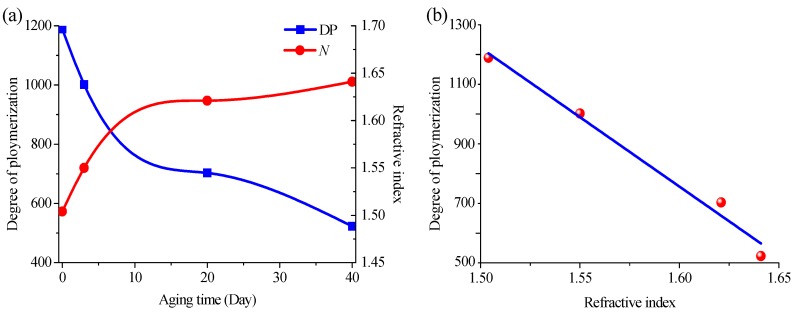
(**a**) Curves of refractive index and degree of polymerization (DP) of insulation paper; (**b**) DP vs. refractive index of paper.

Furthermore, the degree of polymerization (DP) of insulation paper was measured by the conventional method, and the results were similar to Emsley’s findings [28]. Figure 4a shows the curve of the refractive index and the DP with aging time. In this work, a least-squares fit (R^2^ = 0.976) was used to analyze the DP vs. refractive index, which was shown in Figure 4b. It found that there is a good linear relationship between the DP and refractive index of paper.

### 3.3. Molecular Geometric Configuration

**Figure 5:**
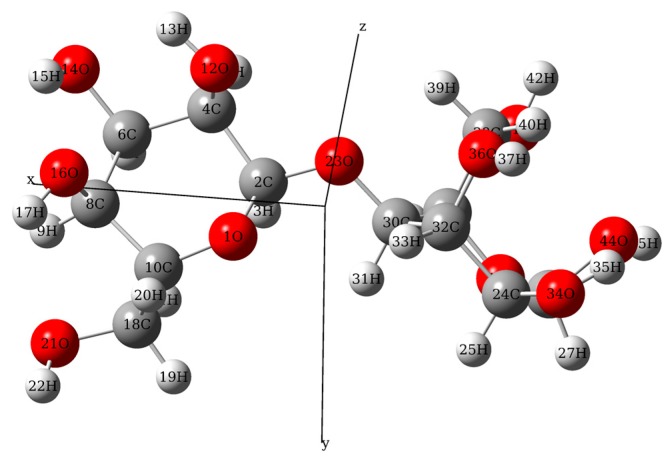
The molecular geometric configuration of the isolated cellobiose molecule.

The main component of the transformer insulation paper was α-cellulose, with a cellobiose (C_12_H_22_O_11_) repeating unit. It is well known that DFT has great advantages in geometric optimization of molecular structure. Therefore, to analyze the paper spectrum, a quantum chemical calculation of cellobiose was performed via Gaussian software (Gaussian Inc., Wallingford, CT, USA). The geometry structure of the isolated molecule of cellobiose was optimized using the hybrid functional model of Becke, three-parameter, Lee-Yang-Parr (B3LYP) with 6-311G+ (d, p) basis set in Gaussian software. The optimized molecular structure was drawn in GaussView (Gaussian Inc., Wallingford, CT, USA). And Figure 5 shows the calculated molecular structure in atomic coordinates. There was no imaginary frequency in the calculation, which means that the obtained molecular conformation was stable. And the atomic coordinate can be used as input for calculating vibration modes.

### 3.4. Comparison of Experimental and Theoretical Spectra

**Figure 6:**
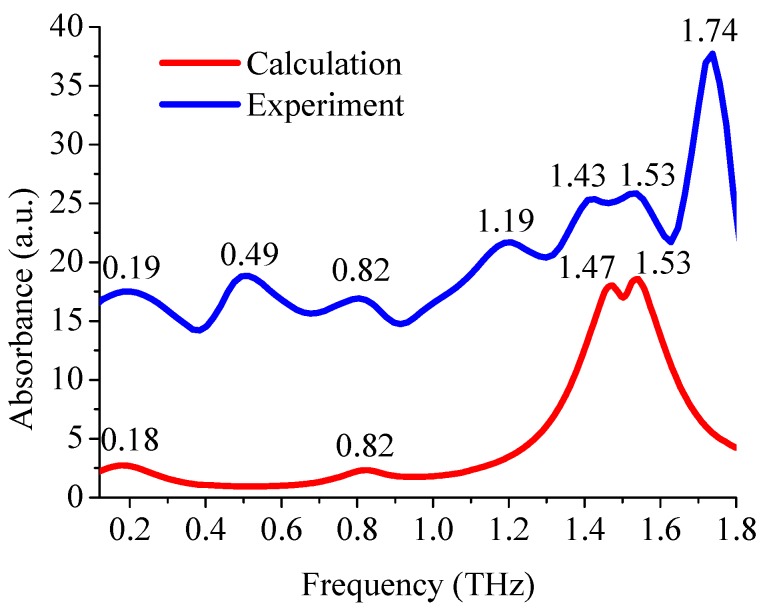
Comparison of theoretical and experimental spectra of the paper (the experimental spectra vertically displaced by 10 a.u.).

The theoretical absorption spectra of isolated cellobiose molecules were simulated based on the calculated atomic coordinates and the optimized molecular structure. Figure 6 shows the comparison between the theoretical spectra simulated by the DFT of B3LYP/6-311G+ (d, p) and the experimental spectra processed by the wavelet algorithm. The calculations revealed absorption peaks at 0.18 THz, 0.82 THz, 1.47 THz, and 1.53 THz, which were in very close agreement with partial experimental absorption peaks. However, there were still some differences, which was mainly due to the different state of the tested sample. Because the experimental sample was in the state of solid sheet, while the simulation was based on the isolated molecule. Therefore, intermolecular interactions and crystal vibrations were not included in the theoretical simulations. In addition, the experiment was carried out at room temperature (296 K) and the simulation was based on the temperature of 0 K, so the thermal effect was ignored. The above results lead to simulated absorption peaks that were less than that of the experiment.

### 3.5. Assignment of Absorption Peaks

**Figure 7:**
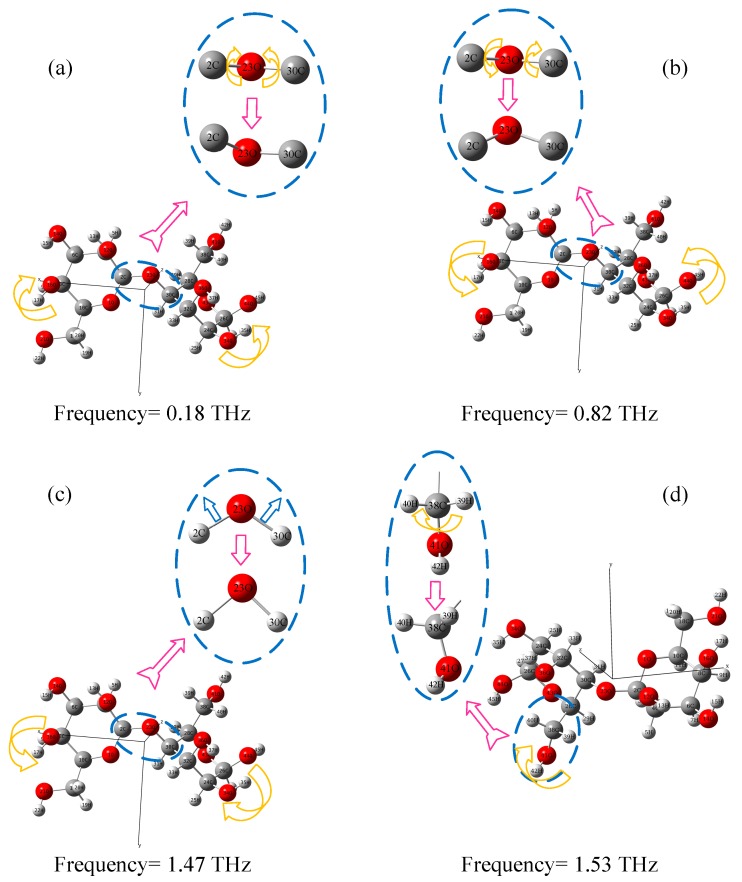
The assignment of simulated absorption peaks of insulation paper. (**a**) peak at 0.18 THz; (**b**) peak at 0.82 THz; (**c**) peak at 1.47 THz; (**d**) peak at 1.53 THz.

The formation mechanisms of THz characteristic absorption peaks assigned and analyzed by GaussView software are shown in Figure 7. It can be explained that the 0.18 THz peak was attributed to an asymmetric bending vibration of the C-O-C (2 C, 23 O and 30 C) group along the positive x-axis in the x-z plane. The 0.82 THz peak was attributed to C-O-C (2 C, 23 O and 30 C) asymmetric bending along the negative x-axis in the x-z plane. The 1.47 THz peak was attributed to C-O-C (2 C, 23 O and 30 C) symmetrical stretching in the x-z plane toward the negative z-axis. The 1.53 THz peak was attributed to a rocking vibration of the C-O (38 C and 40 O) along the x-axis in the x-z plane. The 0.49, 1.19, and 1.74 THz peaks may be generated by intermolecular interactions or crystal vibrations, which could not be simulated by atomic-force Gaussian software.

## 4. Conclusions

In this paper, the THz-TDS was used to study the spectral characteristics of insulation paper after thermal aging intervals in the range of 0.1~1.8 THz. To eliminate spectral noise and improve spectral SNR, the signal was processed by wavelet algorithm. Furthermore, the density function theory (DFT) of B3LYP/6-311G+ (d, p) was applied to the geometric configurations and dynamics simulation of isolated cellobiose molecule. The result shows that there was a good agreement between experimental and theoretical simulation spectra, and the formation mechanism of the absorption peaks was identified and assigned according to the theoretical simulation results. In addition, it is found that there is a good linear relationship between the DP and refractive index of insulation paper by fitting with the least squares fitting, which means that DP can be estimated by measuring the refractive index of insulation paper. Overall, this work proves that THz technology could be used to detect the condition of transformer insulation paper after aging, and helped to establish a mathematical model between THz spectral parameters and aging.
